# Giant liposarcoma of the back with 4 types of histopathology: a case report

**DOI:** 10.1186/1757-1626-2-9339

**Published:** 2009-12-16

**Authors:** Panoraia Paraskeva, Paraskevas Katsaronis, Eleftherios D Spartalis, Andreas C Lazaris, Hara Gakiopoulou, Panagiotis Mallis, Periklis Tomos

**Affiliations:** 1 2nd Department of Propedeutic Surgery, National and Kapodistrian University of Athens, Medical School, 17 Agiou Thoma Str., 115 27, Athens, Greece; 2 1st Department of Pathology, National and Kapodistrian University of Athens, Medical School, 75 Mikras Asias Str., 115 27, Athens, Greece; 3 ENT Department, "Laiko" General Hospital of Athens, 17 Agiou Thoma Str., 115 27, Athens, Greece

## Abstract

The incidence of soft tissue tumours, both malignant and benign, is very common. However, the coexistence of 4 types of histopathology is rare and the aim of this article is to present one treated in our Department. An 87-year-old Greek man was treated in our Department for a huge tumour on his back, under local anaesthesia. The pathology report of the specimen referred 4 types of neoplasia. This case represents this incidence in a giant liposarcoma of the back.

## Introduction

Soft tissue tumours are very common in the general population. Most of them are benign and they require excision for functional and appearance reasons. In literature the incidence of 4 types of histopathology in a specimen is very rare[1] and our aim is to present one.

## Case presentation

**Figure 1:**
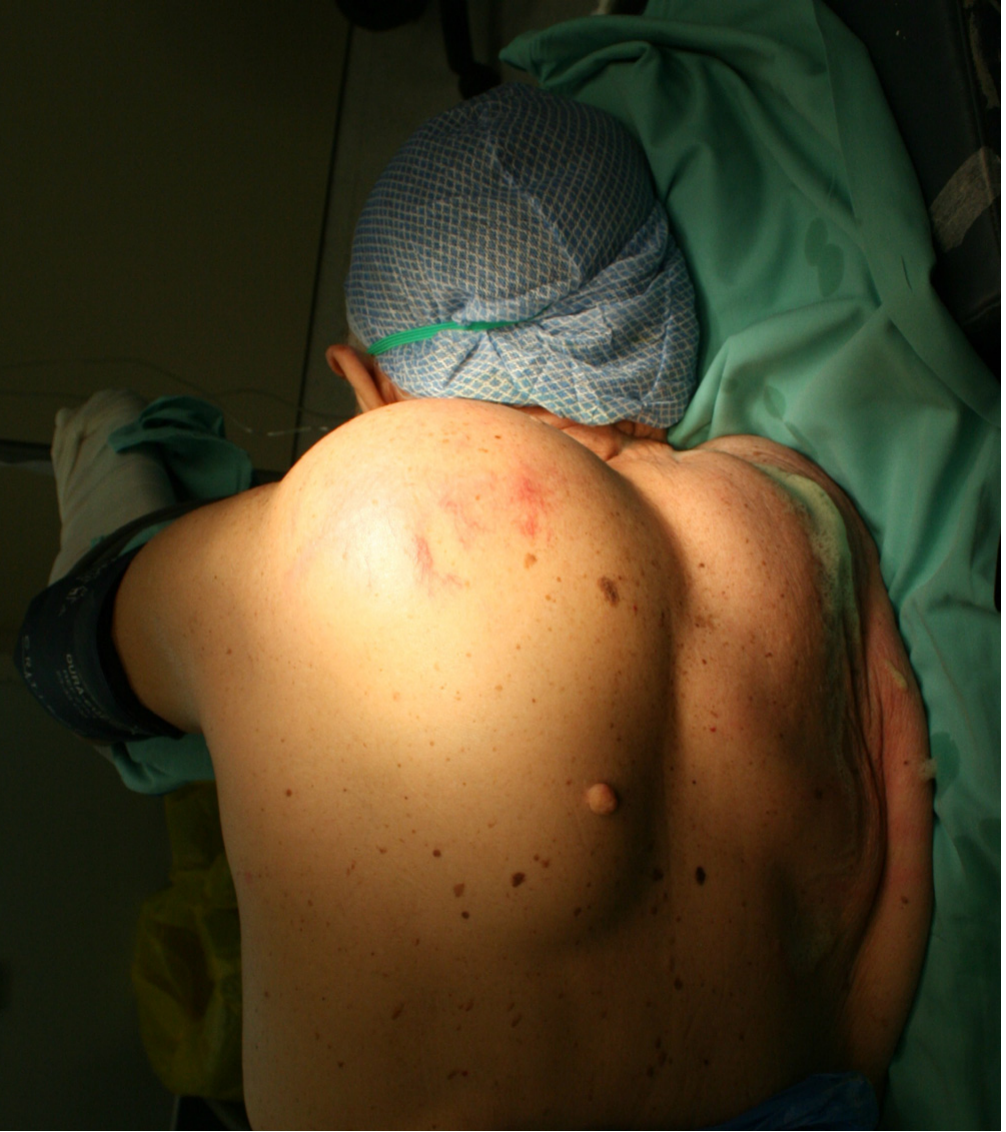
Preoperative image of the patient

**Figure 2:**
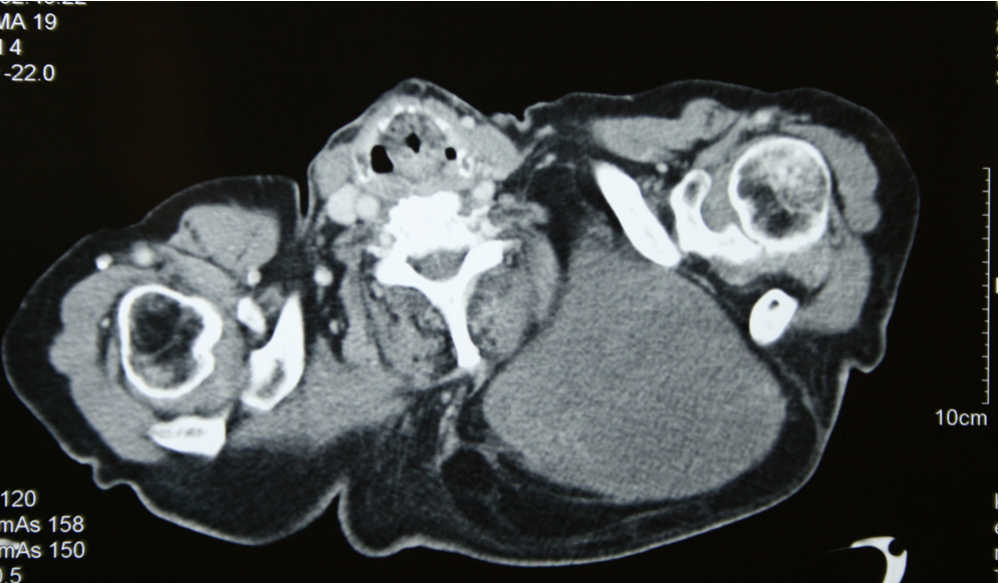
Chest CT scan of the patient

An 87-year-old Greek man, who had been generally active and his past medical history was free of disease, was referred to our hospital for a giant swelling on his back. He has had this tumor since at least 10 years, but he had not undergone an operation as it has been asymptomatic (Fig. 1). Due to the dimensions of the tumor, the patient has undertaken a chest computer tomography scan in order to determine the borders and the invasion of the tumor to the surrounding tissues (Fig. 2)

**Figure 3:**
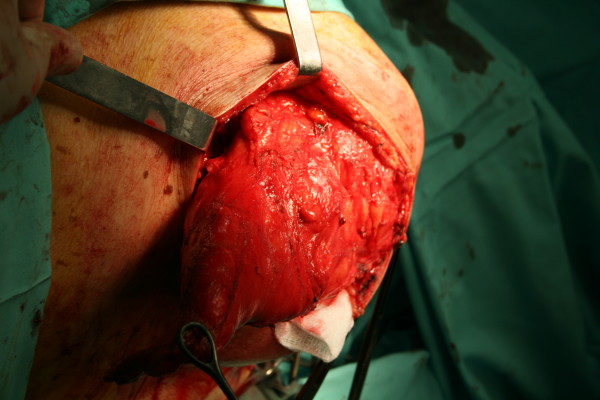
The tumor during the operation

**Figure 4:**
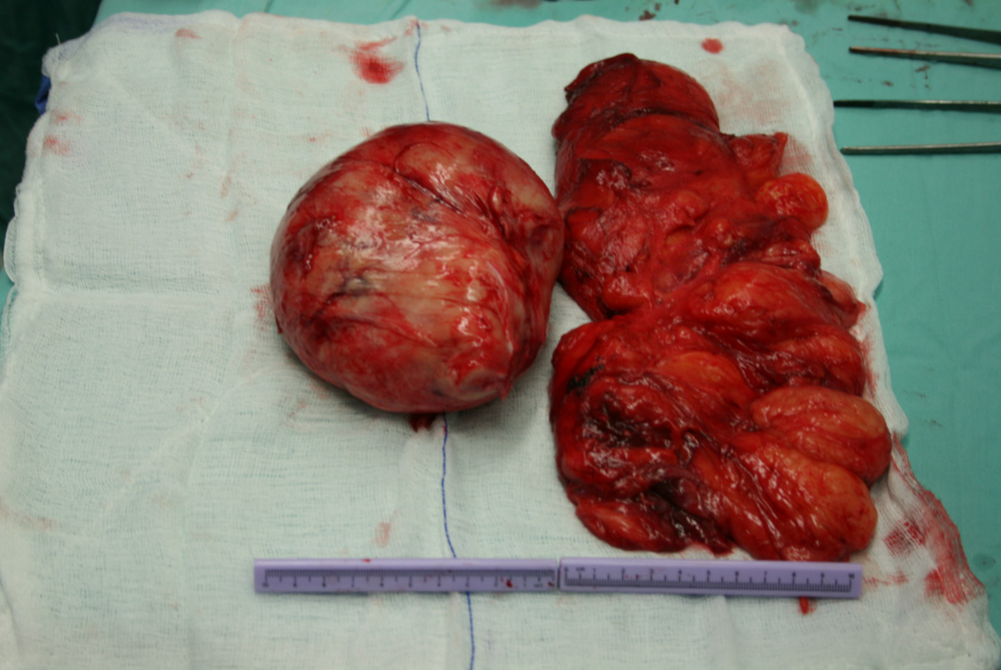
The actual size of the tumor

**Figure 5:**
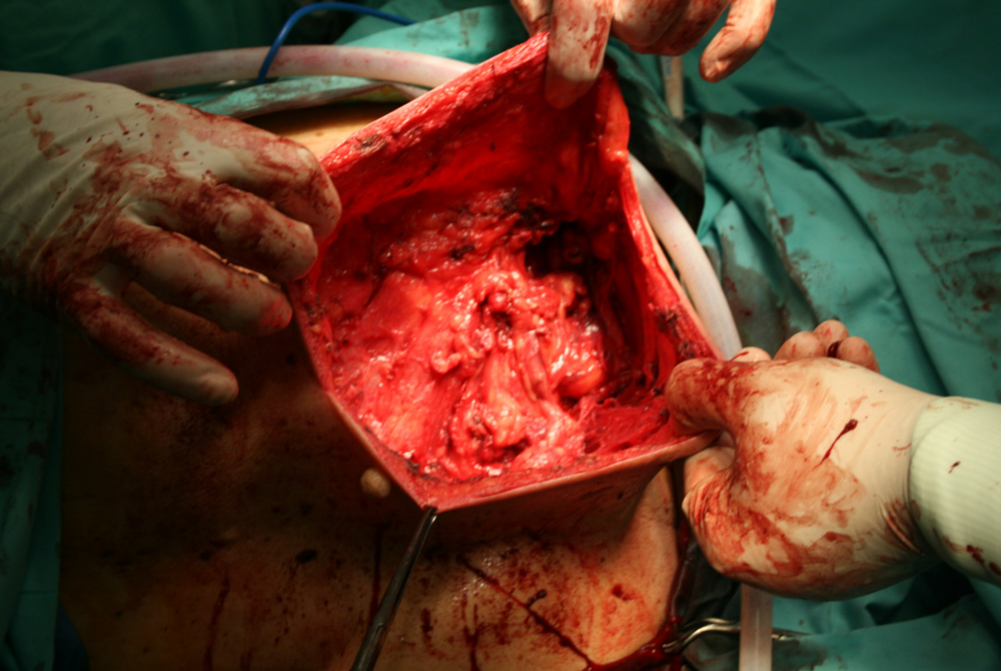
The area of the removed lesion

The patient was referred for a surgical removal of the tumor. Due to his age, he has not taken general anesthesia and the whole operation has been under regional anesthesia. Intraoperatively, a giant liposarcoma, 18 × 14 × 6 cm, was found and been removed and also another tumor, 12 × 10 × 8 cm, white and homogenous (Fig. 3, 4, 5).

**Figure 6:**
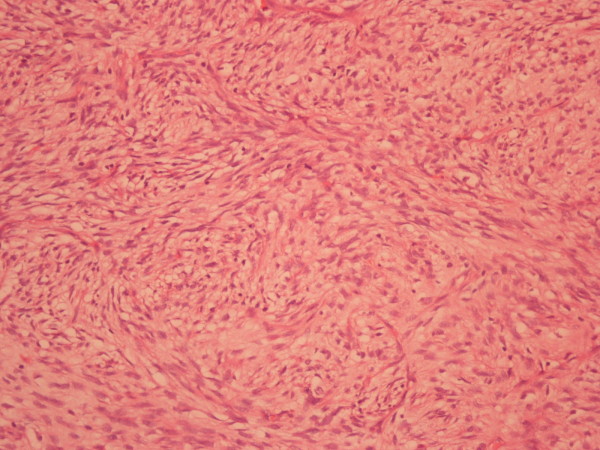
Growth of well differentiated liposarcoma (x 100) hematoxylin and eosin staining

**Figure 7:**
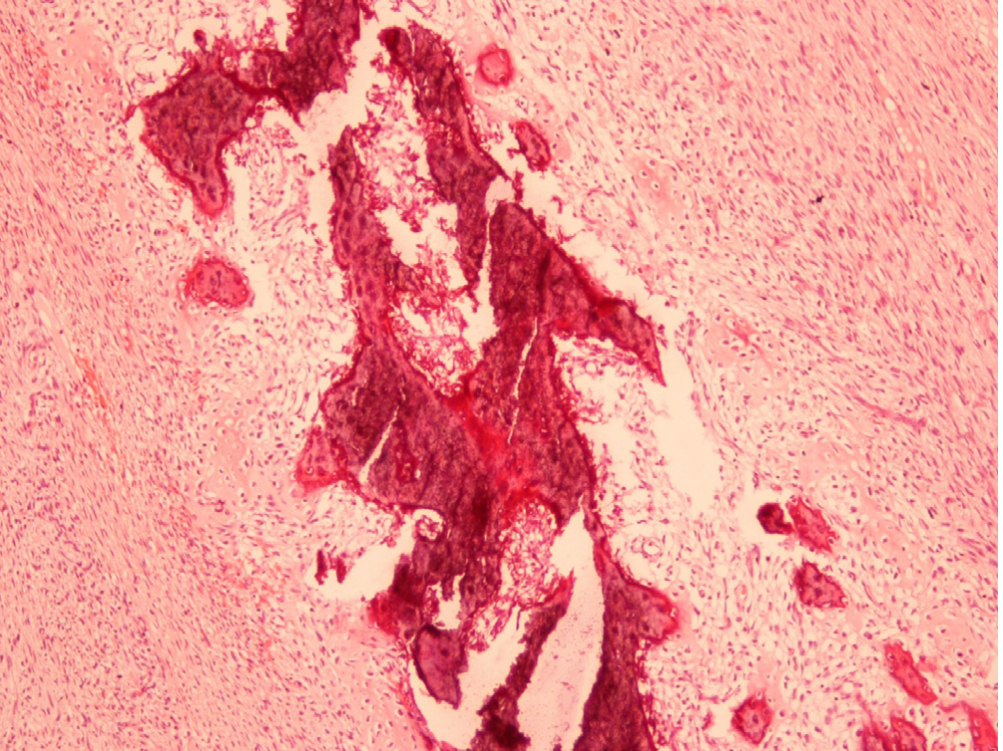
Non-differentiated part of the tumor with the pattern of malignant fibrous histiocytoma (x 100) hematoxylin and eosin staining

**Figure 8:**
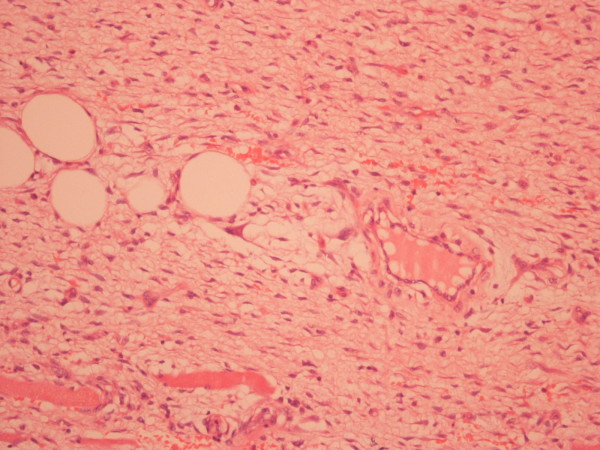
Part of osteosarcoma (X 200) hematoxylin and eosin staining

**Figure 9:**
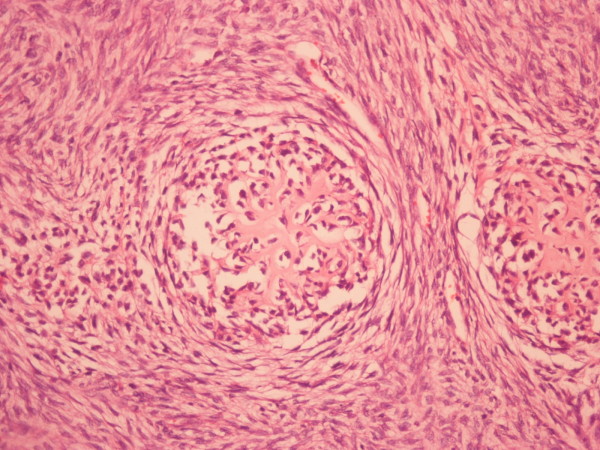
Part od chondrosarcoma (x 100) hematoxylin and eosin staining

The pathology report referred 4 types of neoplasia. There was growth of mesenchymal neoplasma with figure of non-differentiated liposarcoma. There was also growth of well-differentiated liposarcoma (Fig. 6), whereas in the non-differentiated parts of the tumor malignant fibrous histiocytoma (Fig. 7), osteosarcoma (Fig. 8), chondrosarcoma (Fig. 9) and parts with pericellular pattern were found. The skin segment was soft fibroma.

The perioperative period was uneventful and the patient was discharged home 3 days later.

## Discussion

The existence of 4 histological types of neoplasia is very rare and was treated surgically under local anesthesia. Preoperative evaluation of such tumours is essential in order to determine the borders and characterize the tumour [2]. This can be done either with CT scan or MRI scan. The definite diagnosis is set by histology and requires careful analysis of the specimen.

## Competing interests

The authors declare that they have no competing interests.

## Authors' contributions

PP analyzed and interpreted patient's files and wrote the paper. PK and ES contributed in the writing and also assisted at the operation. AL and HG performed the histological examination. PM assisted at the operation and looked after the patient postoperatively. TP was the responsible professor for the patient, decided and performed the operation.

All authors read and approved the final manuscript.

## Consent

Written informed consent was obtained from the patient for publication of this case report and accompanying images. A copy of the written consent is available for review by the Editor-in-Chief of this journal.
